# LSPR Biosensing Approach for the Detection of Microtubule Nucleation

**DOI:** 10.3390/s19061436

**Published:** 2019-03-23

**Authors:** Keisuke Hasegawa, Otabek Nazarov, Evan Porter

**Affiliations:** Department of Physics, Grinnell College, 1116 Eighth Avenue, Grinnell, IA 50112, USA; nazarovo@grinnell.edu (O.N.); porterev17@grinnell.edu (E.P.)

**Keywords:** microtubule nucleation, localized surface plasmon resonance, gold nanoparticles, optical biosensors

## Abstract

Microtubules are dynamic protein filaments that are involved in a number of cellular processes. Here, we report the development of a novel localized surface plasmon resonance (LSPR) biosensing approach for investigating one aspect of microtubule dynamics that is not well understood, namely, nucleation. Using a modified Mie theory with radially variable refractive index, we construct a theoretical model to describe the optical response of gold nanoparticles when microtubules form around them. The model predicts that the extinction maximum wavelength is sensitive to a change in the local refractive index induced by microtubule nucleation within a few tens of nanometers from the nanoparticle surface, but insensitive to a change in the refractive index outside this region caused by microtubule elongation. As a proof of concept to demonstrate that LSPR can be used for detecting microtubule nucleation experimentally, we induce spontaneous microtubule formation around gold nanoparticles by immobilizing tubulin subunits on the nanoparticles. We find that, consistent with the theoretical model, there is a redshift in the extinction maximum wavelength upon the formation of short microtubules around the nanoparticles, but no significant change in maximum wavelength when the microtubules are elongated. We also perform kinetic experiments and demonstrate that the maximum wavelength is sensitive to the microtubule nuclei assembly even when microtubules are too small to be detected from an optical density measurement.

## 1. Introduction

Microtubules (MTs), hollow protein filaments consisting of polymerized α/β-tubulin subunits, are involved in a number of biological processes including cell division, intracellular transport and cell motility. One of the remarkable properties of MTs is the assembly-disassembly dynamics. The end of a MT is stable when it is capped by subunits whose β-tubulins are bound to GTP, and it can elongate by incorporating additional GTP-bound subunits at the end. Once incorporated, GTP is hydrolyzed to GDP, which destabilizes the MT. Loss of the GTP cap exposes unstable GDP-tubulin subunits at the end, and results in MT disassembly. This allows a MT to cycle back and forth between phases of growth and shrinkage, a phenomenon named dynamic instability [1], and this dynamic behavior is critical to the proper functions of MTs. 

Various experimental techniques have been developed to study MT dynamics. One of the most commonly used method for monitoring MT dynamics *in vitro* is based on turbidity or optical density (OD) measurement. The turbidity of a MT sample is generally assumed to be linearly dependent on the concentration of polymerized tubulin subunits, and MT assembly and disassembly can be monitored by recording OD of a sample over time with a spectrophotometer [2]. Other bulk assays based on sedimentation, viscosity and fluorescence-intensity measurements have also been employed to study MT formation *in vitro* [3,4]. Because of their bulk nature, these methods report on the total amount of polymerized tubulin subunits within a given sample. In contrast, electron and optical microscopy, which allow visualization of MTs, have been used to study the dynamic behavior at the individual MT level. For example, fluorescence microscopy is now routinely utilized to track the end dynamics of individual MTs in live cells and *in vitro* [5,6]. Recently, a microscopy method based on fluorescence lifetime imaging (FLIM) measurements of Förster resonance energy transfer (FRET) has also been developed for measuring the amount of polymerized subunits on pixel basis *in vivo* [7].

Although these techniques have helped to improve our understanding of MT dynamics and how it is altered by various microtubule-associated proteins (MAPs) and tubulin-binding compounds, there are still some aspects of MT dynamics that are not well understood. One such aspect is nucleation or the initial formation of a small seed of MT from which the filament can grow from [8]. MT nucleation is the rate-limiting step of the assembly, which determines where and when MTs form inside cells. Currently, there is a limited number of techniques available for investigating MT nucleation. Certain information about the nucleation process can be inferred from turbidity measurements [9,10]. However, because turbidity is insensitive to the length distribution [2], it does not allow for the direct monitoring of the formation of small MT nuclei. Due to the diffraction limit, optical microscopy cannot resolve the interactions of tubulin subunits (each α/β-tubulin heterodimer is roughly 5 nm wide and 8 nm long), which lead to the formation of nascent MTs. Recently, FRET spectroscopy has been used for detecting MT pre-nucleation but under conditions that prevent the formation of MTs [11]. Fluorescence intensity measurement under a total internal reflection fluorescence (TIRF) microscope has allowed the direct observation of templated nucleation from MT seeds stabilized with slowly hydrolyzable GTP analog, guanosine-5′-[(α,β)-methyleno]triphosphate (GMPCPP) [12]. TIRF microscopy has also been used to observe the generation of growing MTs as a way to quantify nucleation from γ-tubulin ring complex (γ-TuRC) *in vitro* [13]. A similar approach using confocal microscopes has been employed to study MT nucleation live cells [14]. However, many of these microscopy-based approaches require specialized experimental setups that are not always readily available. Moreover, because of the stochastic nature of nucleation, the time courses of hundreds of MTs must be analyzed in order to obtain enough statistics. Development of simpler experimental techniques that report on MT nucleation below the diffraction limit could help advance our understanding of the process. 

Here, we report the development of a novel localized surface plasmon resonance biosensing approach for detecting MT nucleation *in vitro*. Surface plasmon resonance (SPR) is a collective resonant oscillation of the free electrons of a metal. When excited, SPR enhances the electric field near the metal-dielectric interface, rendering it highly sensitive to the optical response of the local environment surrounding the metal. SPR sensors take the advantage of this sensitivity to achieve label-free detection of chemical and biological molecules in a sample [15,16]. In a typical affinity-based SPR sensing experiment, a metal surface with immobilized ligands is exposed to a medium containing analytes. The analyte-ligand interaction increases the concentration of the analytes near the metal surface, causing a change in the local refractive index. This, in turn, alters the characteristics of SPRs including the conditions for the excitation of SPRs (e.g., angle of incidence and excitation wavelength) supported on the metal surface, which can be monitored experimentally. SPR sensors based on flat metal surfaces are commercially available and have been used to detect a number of biological analytes and to measure the affinity and kinetics of biomolecular interactions [17]. Biosensors based on SPRs on metal nanoparticles, known as localized surface plasmon resonances (LSPRs), have also been developed to study biomolecular interactions such as antibody-antigen and biotin–streptavidin interactions (see [18,19,20] and references therein) as well as protein aggregations [21]. In a typical LSPR experiment, an increase in the local refractive index causes the extinction spectrum maximum *λ*_max_, which corresponds to the excitation of LSPR, to shift to a longer wavelength. Such a redshift can be observed using a spectrophotometer. One of the key advantages of LSPRs over SPRs on flat metal surfaces is the degree of field confinement. Whereas the electric fields of SPRs extend a few hundred nanometers from a flat metal surface, the electric fields of LSPRs are confined within a few tens of nanometers from the nanoparticles, giving them smaller sensing volumes [22,23]. Furthermore, since the field confinement depends on the size and the shape of nanoparticles, the sensing volume can be tuned geometrically to match the size of analytes [22]. To the best of our knowledge, SPR- or LSPR-based detection of MT or other biopolymer nucleation has not been demonstrated.

The biosensing approach presented here utilizes the geometrically tunable, localized sensing capabilities of the LSPR of gold nanoparticles (AuNPs) to detect MT nucleation. We present a Mie theory-based model for the optical response of AuNPs when MTs form around the nanoparticles, and analyze how the extinction spectrum maximum *λ*_max_ depends on the degree of MT formation. The model also serves as a guide for choosing the size of AuNPs for optimal sensing. To demonstrate MT nucleation sensing experimentally, we induce MT formation from AuNPs with immobilized tubulin, and show that the spectral position of *λ*_max_ is sensitive to the initial formation of MTs but insensitive to their elongation. We also perform a kinetic experiment and show that LSPR allows a real-time sensing of MT nucleation around AuNPs even when the nuclei are too small to be detected with an OD measurement. 

## 2. Materials and Methods

### 2.1. Theoretical Modeling

**Figure 1:**
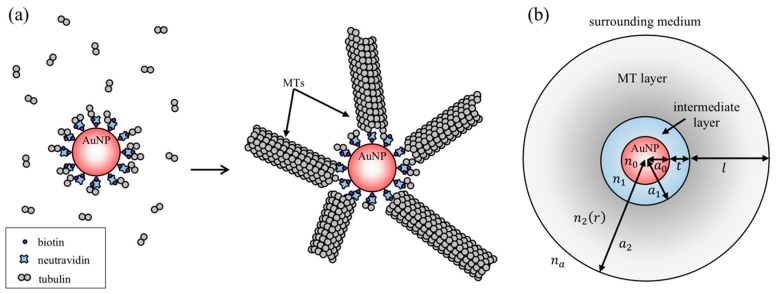
(**a**) A schematic diagram illustrating microtubule (MT) formation around a gold nanoparticle (AuNP), and (**b**) a model used to calculate the extinction spectrum of an ensemble of the MT-bound AuNPs.

To compute the ensemble extinction spectrum of AuNPs bound to MTs in suspension (Figure 1a), we treated each particle to consist of three layers: a spherical gold core with radius *a*_0_, an intermediate layer containing neutravidin, biotinylated tubulin and other factors needed to link MTs to the metal core, and an outer MT layer. The intermediate layer could contain molecules such as polyethylene glycol (PEG) and bovine serum albumin (BSA) for reducing nonspecific binding of proteins to the gold core. We assumed that the intermediate layer has a uniform thickness *t* with effective refractive index *n*_1_ and the MT layer has a uniform thickness *l* (corresponding to the average MT length) with effective refractive index *n*_2_ (Figure 1b). Similar approaches have been employed in the past to study light scattering by protein-coated AuNPs [24,25,26]. A theoretical study using the discrete dipole approximation method has also shown that the effective medium approximation can accurately model a partially coated protein layer on metal nanoparticles [27]. For the surrounding medium, we used the index of refraction of water *n_a_* = 1.33. The refractive index of gold *n*_0_ was taken from a previous work by Johnson and Christy [28]. Because of the spherical geometry of the system and the long persistence length of MTs [29], the density of MTs decreases as *r*^−2^ from the outer surface of the intermediate layer where *r* is the radial distance from the center of the gold core. To account for this variation in our model, we used the Gladstone–Dale relation [30] and assumed that *n*_2_ − *n_a_* is proportional to the local average density of MTs. This allowed us to express the refractive index of the MT layer as:

(1) $$n_{2}\left(r\right)=n_{a}+\Delta n\;\frac{a_{1}^{2}}{r^{2}}$$

where Δ*n* is proportional to the refractive index increment and the number of MTs attached to the nanoparticle, and *a*_1_ is the radial distance from the center of the gold core to the outer surface of the intermediate layer. This expression was based on the assumption that MTs can only grow from the intermediate layer. We excluded the possibility for branching MT nucleation, which could amplify the number of MTs [31]. The protein refractive index increment of 1.90 × 10^−4^ mL/mg [32] was used to estimate *n*_1_ and Δ*n*. We utilized a modified Mie theory that was developed previously to describe light scattering and absorption by a spherical particle with radially variable refractive index [33] and wrote a Mathematica code to compute the extinction spectrum numerically (see Appendix A for details). For bare AuNPs and particles without MT layer, extinction spectra were obtained using the Mie theory for a homogeneous spherical particle [34] and a sphere with a concentric spherical shell [35]. 

### 2.2. Sample Preparation

Porcine tubulin was purified through two cycles of polymerization-depolymerization as outlined by Castoldi and Popov [36]. Biotinylated tubulin and Rhodamine-labeled tubulin were purchased from Cytoskeleton, Inc. (Denver, CO). Tubulin solutions were centrifuged at 200,000× *g* for 10 min at 4 °C to remove tubulin aggregates immediately before use. 

To prepare paclitaxel-stabilized samples, 200 pM of 80 nm biotin-PEG AuNPs (Cytodiagnostics, Inc., Ontario, Canada) was mixed with 520 nM neutravidin (Thermo Fisher Scientific, Waltham, MA, USA) in BRB80 (80 mM PIPES, 1 mM MgCl_2_, 1 mM EGTA, pH 6.8) containing 0.025% Tween 20 (Sigma-Aldirch, St. Louis, MO, USA). A small amount (30 nM) of Atto655-streptavidin (Sigma-Aldirch, St. Louis, MO, USA) was also added to help visualize AuNPs under a fluorescence microscope. To prevent AuNPs from aggregating, neutravidin and Atto655-streptavidin were added in excess to saturate the accessible biotin on AuNPs. The mixture was incubated for 2 h at 4 °C. We decided not to wash off excess neutravidin and Atto655-streptavidin because centrifugation caused AuNPs to aggregate. After the 2 h incubation, an equal volume of 44 µM tubulin solution containing 4% Rhodamine-labeled tubulin and 7% biotinylated tubulin was added and incubated for 1 additional hour at 4 °C. To induce MT formation, 1/10 volume of 10 mM GMPCPP (Jena Bioscience, Thuringia, Germany) was added and incubated at 37 °C for 30 min. For samples with short MTs, 20 volumes of BRB80 supplemented with 10 µM paclitaxel (Sigma-Aldirch, St. Louis, MO, USA) was added to stabilize MTs. For samples with longer MTs, 10 volumes of 4 µM tubulin solution containing 4% Rhodamine-labeled tubulin and 0.5 mM GMPCPP was added and incubated for another 20 min at 37 °C before paclitaxel was introduced to stabilize MTs. Here, the concentration of the additional tubulin introduced was kept low to promote MT elongation from existing MTs while limiting new MTs from nucleating. Samples were stored at room temperature after the addition of paclitaxel, and were processed for fluorescence microscopy and ultraviolet-visible spectroscopy within 2 h of sample preparation during which there was no visible change in MT length. As a negative control, we also prepared a sample without MT formation by following the same procedures as those for short MT samples except we did not add GMPCPP and it was not incubated at 37 °C. 

To prepare samples for kinetic experiments, 70 pM of 80 nm biotin-PEG AuNPs were incubated with 340 nM neutravidin in BRB80 containing 0.025% Tween 20 for 2 h at 4 °C. To reduce non-specific interaction with tubulin, AuNPs were blocked with 1/4 volume of 5 mg/mL BSA (Sigma-Aldirch, St. Louis, MO, USA) for 1 h. An equal volume of 22 µM tubulin solution containing either 0% or 7% biotinylated tubulin was added and the mixture was incubated for 1 h at 4 °C before 1/10 volume of 5 mM GMPCPP was added. Samples were kept on ice until they were ready to be placed in a spectrophotometer equipped with a temperature-controlled cell for collecting extinction spectra. 

### 2.3. Fluorescence Microscopy

Samples were imaged with a 60 × 1.40 NA plan-apochromat oil immersion objective lens on an Olympus XI-81 inverted microscope. Images were acquired using a Hamamatsu ORCA-ER CCD camera (Hamamatsu Photonics, Hamamatsu, Japan) and SlideBook 6 software (Intelligent Imaging Innovations, Inc, Denver, CO, USA). To promote the attachment of microtubule samples to the glass substrate, coverslips were immersed in 0.1 mg/mL poly-L-lysine (Sigma-Aldirch, St. Louis, MO, USA) for 2 h, washed with dH_2_O, and air dried before use. 

### 2.4. Ultraviolet-Visible Spectroscopy and Data Analysis

Extinction spectra were collected using a Cary 60 Spectrophotometer equipped with a single cell Peltier temperature controller (Agilent Technologies, Inc., Santa Clara, CA, USA) and quartz cuvettes (FireflySci, Inc., Staten Island, NY, USA). For paclitaxel-stabilized samples, spectra were collected with 1 nm step and scan speed of 600 nm/min at room temperature. For kinetic experiments, the extinction spectra before MT formation were first collected by keeping the temperature of the Peltier cell holder at 4 °C and with scan speed of 2400 nm/min. The samples were then placed on ice while the temperature of the holder was increased to 32 °C. Once the temperature reached 32 °C, the samples were placed in the spectrophotometer and the spectra were acquired every 30 s. The first time point was excluded from analysis because condensation on the cuvette surface made significant contributions to the extinction spectra, rendering it unusable for measuring the peak wavelength. BRB80 was used as the baseline.

To determine the extinction maximum wavelength (*λ*_max_), we used Mathematica to interpolated each spectrum by fitting a polynomial of degree 10 from 500–610 nm, which approximately spans the full width at half maximum (FWHM) of the LSPR peak. The purpose of the polynomial fitting was to reduce noise and track the peak position below the wavelength resolution of the spectrophotometer, and not to fit the spectrum to a physical model. A similar approach based on calculating the centroid wavelength (*λ*_centroid_) had been used to improve the spectral resolution of LSPR sensors [37,38]. For the present study, we chose to track *λ*_max_ instead because we found *λ*_centroid_ to be noisier possibly due to its sensitivity to a slight variation in the width of the resonance. The original interpolated centroid-tracking algorithm was based on the assumption that the width of a resonance would not change [39], which was not always the case in our study. Statistical analyses were performed using GraphPad Prism and Excel. 

## 3. Results and Discussion

### 3.1. Theoretical Modeling

To investigate how the formation of MTs around AuNPs affect the characteristics of LSPRs, we developed a Mie theory model of the nanoparticle system illustrated in Figure 1b, and calculated how the extinction spectrum depends on the MT layer thickness. The purpose of our theoretical work was not to build a physically precise model of the nanoparticles with detailed descriptions of bound MTs (such as the number of MTs on each AuNP, their positions, and length distribution) from which the extinction spectrum can be calculated. Instead, the model was developed as a first-order approximation tool that can be used to understand how the sensing volumes of the nanoparticles depend on various physical parameters and to predict how the extinction spectrum maximum *λ*_max_ depends on the degree of MT formation. 

**Figure 2:**
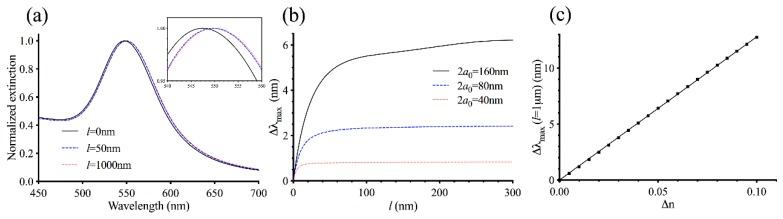
Calculated localized surface plasmon resonance (LSPR) response of 80 nm AuNPs without the intermediate layer. (**a**) Calculated normalized extinction spectra for AuNPs with diameter 2*a*_0_ = 80 nm, *t* = 0, and various MT layer thickness *l*. The inset shows a magnified view of the spectra near their peaks. (**b**) The spectral shift Δ*λ*_max_ = *λ*_max_(*l*) − *λ*_max_(0) as a function of the MT layer thickness for AuNPs with different diameters. (**c**) Plot of Δ*λ*_max_ as a function of Δ*n* for AuNPs with 2*a*_0_ = 80 nm. The solid line shows a linear fit to the calculated data points.

The extinction spectrum depends on three physical parameters: the AuNP diameter (2*a*_0_), the intermediate layer thickness *t*, and the MT layer thickness *l*. Because it was challenging to consider how the spectrum depends on all three parameters at once, we initially considered a simplified system without the intermediate layer by setting *t* = 0 (see Appendix A) and calculated the extinction spectra with different values for the MT layer thickness *l*. Later, we considered a more realistic system with t > 0. Figure 2a shows the spectra for AuNPs with diameter 2*a*_0_ = 80 nm and the MT layer characterized by Δ*n* = 0.02. As *l* increases, the extinction maximum *λ*_max_ redshifts while FWHM remains roughly constant. A relatively large shift in *λ*_max_ is seen between the bare nanoparticle (*l* = 0 nm) and the particle with a MT layer with thickness *l* = 50 nm. In contrast, the wavelength shift between *l* = 50 nm and *l* = 1000 nm is barely visible even though there is a 20-fold increase in the thickness. To explore the sensing volumes of AuNPs further, we calculated Δ*λ*_max_ = *λ*_max_(*l*) − *λ*_max_(0) as a function of the MT layer thickness *l* where *λ*_max_(0) is the extinction maximum for the bare nanoparticle for AuNPs with diameter 2*a*_0_ = 80 nm. As shown in Figure 2b, Δ*λ*_max_ increases with increasing MT layer thickness *l*, and it levels off at Δ*λ*_max_ ≈ 2.5 nm. Below *l* = 50 nm, the slope is relatively steep and Δ*λ*_max_ depends strongly on *l*. Above *l* = 50 nm, the slope is flatter and Δ*λ*_max_ does not change appreciably with *l*. Thus, consistent with previous studies [22,23], our theoretical model indicates that *λ*_max_ is sensitive to the local refractive index within a few tens of nanometers from the nanoparticle surface but insensitive to the refractive index outside this region.

We also investigated how the particle size affect the sensing volume. Decreasing the nanoparticle diameter by a half to 2*a*_0_ = 40 nm reduces the sensing volume significantly as Δ*λ*_max_ begins to level off near *l* = 10 nm (Figure 2b). Such a change is accompanied by a lower signal as Δ*λ*_max_ remains less than 1 nm as *l* increases. On the other hand, doubling the particle size to 2*a*_0_ = 160 nm causes Δ*λ*_max_ to increase steadily well beyond *l* = 50 nm eventually exceeding 6 nm, indicating that it has a larger sensing volume as well as signal. As a result, there is a trade-off between the signal and the LSPR’s specificity for MT nucleation. Larger nanoparticles can exhibit a greater change in *λ*_max_ upon MT formation, which would be easier to detect experimentally. However, because of their large sensing volumes, *λ*_max_ can continue to shift even when the MT layer becomes a couple of hundred nanometers in thickness. Therefore, a part of the spectral shift can be attributed to MT elongation and not just to MT nucleation. Large nanoparticles have another disadvantage. The extinction peak broadens as the particle size increases (Figure S1), and it becomes more challenging to pinpoint the spectral position of *λ*_max_ experimentally. In contrast, smaller nanoparticles do not display as much spectral shift in *λ*_max_, which can be more difficult to measure compared to larger nanoparticles. But because of their smaller sensing volumes, the response of *λ*_max_ to MT formation can be more specific to nucleation. Based on these considerations, we decided to focus our attention on AuNPs with diameter 2*a*_0_ = 80 nm. 

To understand how the density of MTs formed around AuNPs affect the LSPR, we next explored the relationship between Δ*n* and Δ*λ*_max_. According to the Gladstone–Dale relation [30], Δ*n* is proportional to the local protein concentration and having more MTs nucleating in the vicinity of the nanoparticles should create a larger Δ*n*. Because a spectral shift in *λ*_max_ is a result of a local refractive index change, we expect a larger Δ*n* to result in a greater Δ*λ*_max_. To simplify the analysis, we used a large value for the MT layer thickness *l* and computed the maximum Δ*λ*_max_ that can be achieved for each value of Δ*n*. Calculating *λ*_max_ becomes more computationally intensive as the value of *l* increases because the differential equation for the Debye potential must be solved numerically over a larger computational domain (see Appendix A). To make the computation manageable, we chose *l* = 1 µm, which is significantly larger than *l* = 50 nm where the graph of Δ*λ*_max_ vs *l* begins to level off (Figure 2b). Using *l* > 1 µm would have a negligible effect on Δ*λ*_max_ since the sensing volume around each AuNP is essentially completely occupied. Figure 2c shows a graph of Δ*λ*_max_ as a function of Δ*n* for nanoparticles with diameter 2*a*_0_ = 80 nm. Consistent with our expectation, Δ*λ*_max_ increases monotonically with Δ*n*. Importantly, the relationship is linear to a good approximation with a slope of 128 nm/refractive index unit (RIU). Thus, provided that MTs are sufficiently long such that *l* ≳ 50 nm, the degree of Δ*λ*_max_ can be used as a direct measure of the amount of MTs that have nucleated from the nanoparticles. 

**Figure 3:**
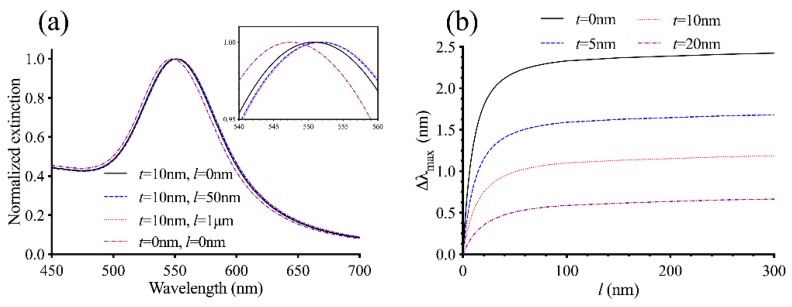
Calculated LSPR response of 80 nm AuNPs with the intermediate layer and *n*_1_ = 1.37. (**a**) Calculated normalized extinction spectra for AuNPs with diameter 2*a*_0_ = 80 nm, intermediate layer with thickness *t* = 10 nm and refractive index *n*_1_ = 1.37, and various MT layer thickness *l*. The inset shows a magnified view of the spectra near their peaks. The spectrum for a bare AuNP (*t* = 0, *l* = 0) is also shown for comparison. (**b**) The spectral shift Δ*λ*_max_ = *λ*_max_(l) − *λ*_max_(0) as a function of the MT layer thickness *l* for nanoparticles with different values for *t*.

So far, we have considered a simplified model without the intermediate layer. To investigate the effect of the intermediate layer on the LSPR, we initially calculated the extinction spectra by assuming that the layer has thickness *t* = 10 nm with refractive index *n*_1_ = 1.37. The diameter of the gold core is set to 2*a*_0_ = 80 nm. To isolate the effect of the intermediate layer and make the comparison with the system considered in Figure 2 straightforward, we adjusted the value of Δ*n* to

(2) $$\Delta n=0.02\times \frac{a_{0}^{2}}{{\left(a_{0}+t\right)}^{2}}$$

so that the refractive index of the MT layer *n*_2_(*r*) is identical for *r* > *a*_0_ + *t* regardless of the value of *t*. Figure 3a shows the normalized extinction spectra for the system with different values for the MT layer thickness *l*. Comparison with Figure 2a indicates that the presence of the intermediate layer causes the extinction spectra to redshift by 2–4 nm with the greatest redshift seen in the spectrum for *l* = 0. Because of this, the difference between the extinction maximum *λ*_max_ for *l* = 0 and that for finite *l* becomes smaller in the presence of the intermediate layer (Figure 3b). This is expected because the intermediate layer occupies a part of the LSPR sensing volume, which would reduce the amount of the MT layer that can be within this volume. As a result, there would be a smaller spectral shift Δ*λ*_max_ upon the formation of MTs. Increasing the intermediate layer thickness from *t* = 10 nm to *t* = 20 nm further lowers Δ*λ*_max_, while reducing the thickness to *t* = 5 nm increases Δ*λ*_max_ (Figure 3b). Using a different value for the layer’s refractive index *n*_1_ results in the same trend (Figure S2). These results indicate that, from a sensor design perspective, it is advantageous to use as thin of an intermediate layer as possible so that the response of *λ*_max_ to MT nucleation can be maximized. If a thick intermediate layer must be used (for example, because of the way MTs are induced to grow from AuNPs), larger AuNPs could be used to compensate for the sensing volume occupied by the layer. Comparison of Figure 3b with Figure S2 also shows that Δ*λ*_max_ as a function of *l* does not depend strongly on the refractive index *n*_1_, and hence, the protein concentration of the intermediate layer. 

### 3.2. Experimental Demonstration of Localized Surface Plasmon Resonance (LSPR) Biosensing Approach for Detecting Microtubule (MT) Nucleation

**Figure 4:**
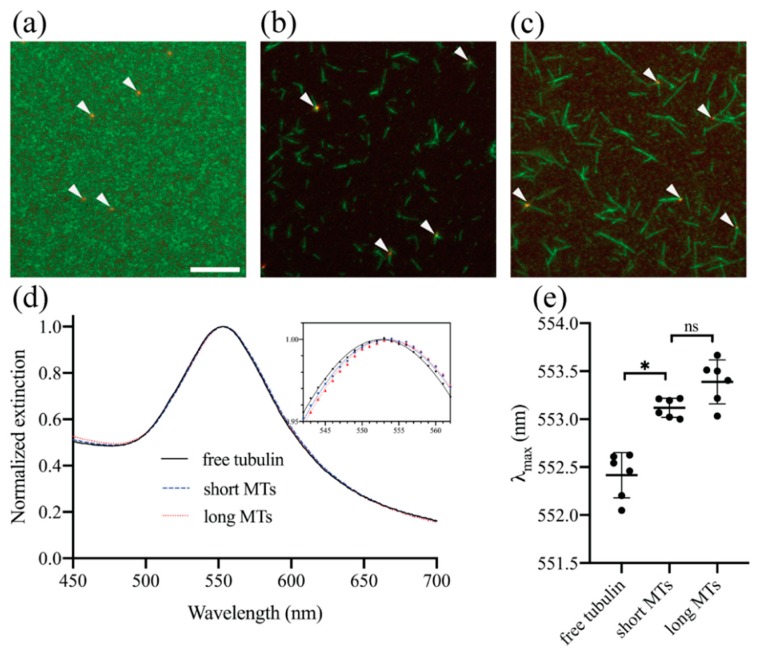
Fluorescence images and extinction spectra of paclitaxel-stabilized samples. Representative fluorescence images of biotin-polyethylene glycol (PEG) AuNPs decorated with neutravidin and ATTO655-streptavidin (red, also indicated with white arrowheads) and tubulin mixture containing biotinylated tubulin and Rhodamine-labeled tubulin (green) (**a**) without GMPCPP and a 37 °C incubation, and (**b**) after a 30-min incubation at 37 °C in the presence of GMPCPP. (**c**) To elongate MTs, a low concentration of additional tubulin was added after the 30-min incubation in (**b**), and the sample was incubated for 20 more minutes at 37 °C. Scale bar, 10 µm. (**d**) Representative, experimentally measured extinction spectra of samples in (**a**)–(**c**). The inset shows a magnified view of the measured spectra (points) near their peaks and corresponding polynomial fits (lines). (**e**) Dot plot of LSPR peak wavelengths from 6 independent experiments. The Wilcoxon matched-pairs signed-rank test was used to calculate the p-values. ∗ *p* < 0.05.

To demonstrate that the LSPR allows the detection of MT nucleation experimentally, we used biotin-neutravidin interactions to immobilize tubulin subunits on 80 nm biotin-PEG AuNPs and induced spontaneous MT formation in the presence of GMPCPP (see Materials and Methods). We hypothesized that by locally concentrating tubulin subunits on the nanoparticle surfaces, we can promote MTs to nucleate from AuNPs. No MTs formed in the absence of GMPCPP and without a 37 °C incubation (Figure 4a). Consistent with our expectation, inducing MT formation by incubating the sample at 37 °C for 30 min in the presence of GMPCPP caused short MTs to form around AuNPs with some of the nanoparticles having multiple MTs around them, reminiscent of small MT asters (Figure 4b). A shorter, 1-min incubation with GMPCPP also resulted in tiny MTs with their ends colocalizing with AuNPs (Figure S3). Together, these results indicate that MTs are nucleating from AuNPs, although we cannot entirely rule out the possibility that some of the MTs became attached to AuNPs shortly after they nucleated away from AuNPs. Not all MTs nucleated from AuNPs as there were also MTs not attached to AuNPs. We were not able to form as many MTs around AuNPs using GTP likely due to its lower nucleating potential compared to GMPCPP. Therefore, we decided to focus our attention on GMPCPP-induced spontaneous MT formation. We also prepared a sample with longer MTs by introducing a low concentration of additional free tubulin after the 30-min incubation in Figure 4b and incubating for 20 more minutes at 37 °C (Figure 4c). 

Figure 4d,e shows the extinction spectra and *λ*_max_ of the free tubulin (Figure 4a), short MT (Figure 4b), and long MT samples (Figure 4c). In the absence of MT formation, AuNPs exhibit LSPR with peak wavelength at *λ*_max_ = 552.4 ± 0.3 nm (mean ± s.d. of 6 independent experiments). The formation of short MTs cause the peak wavelength of redshift to *λ*_max_ = 553.1 ± 0.1 nm, which was statistically significant. In contrast, elongating MTs did not lead to a further redshift that was statistically significant (*λ*_max_ = 553.3 ± 0.2 nm). With the current sensitivity of our technique, we were unable to resolve a slight difference in *λ*_max_ that may exist between short MT and long MT samples. However, our theoretical model (Figure 4) suggests that such a difference if it exists is less than 0.1 nm, much smaller than the observed difference between the free tubulin sample and the short MT sample. Taken together, these results show that consistent with the theoretical model, LSPR is sensitive to the formation of short MTs but insensitive to MT elongation. It should be noted that unlike the calculated extinction spectra, which only include absorption and scattering of light by AuNPs and surrounding protein layers, experimentally measured spectra also include contributions from the absorption of light by ATTO655 and Rhodamine as well as light scattering by MTs that are not attached to AuNPs. However, because their extinction spectra are much smaller than the spectrum of AuNPs (Figure S4), the observed spectra are mostly due to LSPR and the presence of ATTO655, Rhodamine and MTs do not affect *λ*_max_ significantly.

To test if the observed Δ*λ*_max_ upon the formation of MTs agrees with the theoretical model quantitatively, we made the following assumptions to calculate Δ*λ*_max_. We assumed that neutravidin (~5.6 × 5 × 4 nm) [40], biotinylated tubulin (~8 × 5 × 4 nm) and biotin-PEG (10,000 Da, hydrodynamic radius ~3 nm) [41] on the AuNP create an intermediate layer with thickness *t* = 10 nm. Here, we did not take the full dimensions of biotinylated tubulin into account because the presence of a biotinylated subunit at a certain distance from the AuNP surface would not necessarily preclude free subunits to bind to the biotinylated subunit at a similar distance from the nanoparticle as tubulin subunits can bind both longitudinally and laterally. As discussed above, Δ*λ*_max_ does not depend strongly on the refractive index of the intermediate layer, and we assumed *n*_1_ = 1.37 for the purpose of the calculation. We were unable to determine the number of MTs attached to each AuNP accurately from fluorescence images because we could not rule out the possible existence of short, diffraction limited MTs around the AuNPs or MTs that are oriented obliquely to the focal plane. Nevertheless, assuming that on average 3 MTs are attached to each AuNP at their ends and they are oriented perpendicular to the nanoparticle surface, we estimate that on the outer surface of the intermediate layer (surface area 31,400 nm^2^), there are 42 tubulin subunits (14 protofilaments in a GMPCPP-MT) with molecular weight 110 kDa, which corresponds to a protein density of 31 mg/mL or Δ*n* = 0.006 using refractive index increment 1.90 × 10^–4^ mL/mg. From these values, our model predicts a peak wavelength shift of Δ*λ*_max_ = 0.4 nm. Although not exact, this is in reasonable agreement with the observed Δ*λ*_max_ (Figure 4e). 

**Figure 5:**
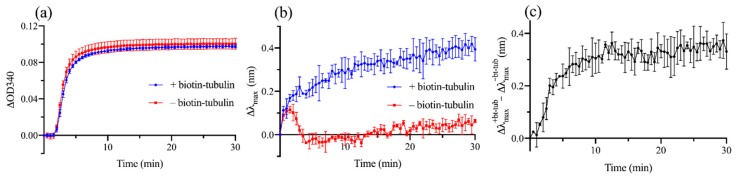
Kinetic experiment results showing changes in optical density at *λ* = 340 nm and *λ*_max_ as functions of time. (**a**) Changes in OD340 and (**b**) Δ*λ*_max_ for samples with and without biotinylated tubulin as functions of time. (**c**) Reference subtracted Δ*λ*_max_ showing the effect of MT formation on the extinction maximum wavelength. The data points show mean ± s.d. of 3 independent experiments.

To further verify that LSPR can detect MT nucleation, we next performed a kinetic experiment and tracked *λ*_max_ in real time as MTs formed. We also measured the optical density at *λ* = 340 nm (OD340) simultaneously as a way to monitor MT formation. A relatively low concentration of tubulin (10 µM, containing 7% biotinylated tubulin) was used to ensure that a lag phase, which corresponds to an initial period of MT nucleus assembly, was clearly visible in the OD340 measurement. A sample without biotinylated tubulin was used as a reference. Figure 5a,b shows changes in OD340 and *λ*_max_ as functions of time *T*. For both the samples with and without biotinylated tubulin, the OD340 measurement showed a lag phase lasting ~2 min followed by a growth phase during which the optical density increased rapidly as nucleated MTs elongate and an equilibrium phase when the concentration of polymerized tubulin subunits reached a steady state. The sample with biotinylated tubulin showed a rapid increase in Δ*λ*_max_ from *T* = 0, approaching Δ*λ*_max_ ≈ 0.4 nm as *T* increased. On the other hand, without biotinylated tubulin, there was no significant change in the peak wavelength except for a temporary increase in Δ*λ*_max_ lasting for the first few minutes of the experiment. Therefore, Δ*λ*_max_ observed in the sample with biotinylated tubulin ($\Delta \lambda_{\max}^{+\mathrm{bt}-\mathrm{tub}}$) can be mostly attributed to the formation of MTs around AuNPs. It is not clear what caused the temporary increase in Δ*λ*_max_ in the sample without biotinylated tubulin ($\Delta \lambda_{\max}^{-\mathrm{bt}-\mathrm{tub}}$), although it could be due to a transient change in the conformation of proteins and PEG molecules bound to AuNPs induced by an abrupt increase in temperature. To isolate the effect of MT formation around AuNPs, we subtracted $\Delta \lambda_{\max}^{-\mathrm{bt}-\mathrm{tub}}$ from $\Delta \lambda_{\max}^{+\mathrm{bt}-\mathrm{tub}}$. The reference subtracted Δ*λ*_max_ (Figure 5c) showed a rapid increase during the first few minutes of the experiment including the lag phase of the OD340 measurement. Taken together, these results demonstrate that LSPR allows a direct detection of MT nucleation around AuNPs even when the nuclei are too small to be detected with OD340, which is consistent with our theoretical model predicting a sub-diffraction-limited sensing volume of LSPR.

## 4. Conclusions

In summary, we have developed a LSPR biosensing approach for the direct detection of MT nucleation *in vitro*. To the best of our knowledge, this is the first study demonstrating that LSPR can be used to monitor MT or other biopolymer nucleation. Using a modified Mie-theory with radially variable refractive index, we built a theoretical model to predict the optical response of AuNPs when MTs form around them and how the extinction maximum *λ*_max_ depends on various parameters such as the AuNP size, the thicknesses of the intermediate layer containing factors needed to link MTs to the gold core, and the MT layer thickness. The model predicted that *λ*_max_ is sensitive to a change in the local refractive index induced by MT nucleation within a few tens of nanometers from the nanoparticle surface, but insensitive to a change in the refractive index outside this region due to MT elongation. More specifically, the model predicted that for 80 nm AuNPs, *λ*_max_ is sensitive to a change in local refractive index within 50 nm from the metal surfaces. The model also predicted that the sensing volume surrounding AuNPs can be tuned geometrically with the nanoparticle size. To demonstrate detection of MT nucleation experimentally, we immobilized tubulin subunits on 80 nm AuNPs and induced spontaneous MT formation in the presence of free tubulin and GMPCPP. Consistent with the theoretical model, we observed an increase in *λ*_max_ upon the formation of short MTs around AuNPs, but no significant change in *λ*_max_ when the MTs were elongated. We also performed a kinetic experiment and showed that *λ*_max_ is sensitive to the assembly of MT nuclei even when they are too small to be detected with the turbidity measurement. 

A unique feature of the approach presented here is that it has a high, geometrically tunable spatial resolution capable of detecting MT nucleation. At the same time, it is bulk-based. Therefore, unlike microscopy-based assays that require analyzing many MTs individually to obtain sufficient statistics, the average behavior of numerous MTs can be obtained from one experiment. Another key advantage is its relative simplicity. It does not require specialized equipment and can be performed using spectrophotometers that are commonly available. A microplate spectrophotometer should enable analysis of multiple samples in parallel. Moreover, because Δ*λ*_max_ is caused by a change in the local refractive index, tubulin subunits do not need to be fluorescently labeled. Yet another advantage of the method is its versatility. Although biotinylated-tubulin subunits were immobilized on AuNPs to detect spontaneous MT nucleation in this work, immobilizing other proteins and protein complexes such as γ-TuRC and TPX2 on AuNPs should allow investigations of MT nucleation from such nucleation templates and factors using LSPR. Additionally, it should be possible to extend the computational model and experimental approach outlined in this article to study nucleation of other biopolymers such as actin filaments. While we were unable to achieve a high signal-to-noise ratio with spherical AuNPs, we expect that using nonspherical nanoparticles such as nanorods and nanotriangles [18] will increase the LSPR sensitivity to MT nucleation. 

## Supplementary Materials

The following are available online at https://www.mdpi.com/1424-8220/19/6/1436/s1, Figure S1: Calculated normalized extinction spectra for AuNPs with diameter 2*a*_0_ = 160 nm, *t* = 0, and various MT layer thickness *l*, Figure S2: Calculated LSPR response of 80 nm AuNPs with the intermediate layer and n_1_ = 1.4, Figure S3: Fluorescence image of paclitaxel-stabilized sample after 1-min incubation at 37 °C, Figure S4: Comparison of the extinction spectra of biotin-PEG AuNPs, neutravidin (Nav) and ATTO655-streptavidin (Sav), free tubulin and MTs containing 4% Rhodamine-labeled tubulin.

## Appendix A

Our approach for calculating for the extinction spectrum of the system shown in Figure 1B is based on the theory of light scattering and absorption by a spherical particle with radially variable refractive index developed by Perelman [33]. Following the notation used by the author, we introduce dimensionless radial parameter *ρ* = *k*_0_*r* where *k*_0_ = 2*π*/*λ* and *λ* is the wavelength of light in vacuum. In terms of *ρ*, the radius of the core is *ρ*_0_ = *k*_0_*a*_0_, the outer radius of the intermediate layer is *ρ*_1_ = *k*_0_*a*_1_ where *a*_1_ = *a*_0_ + *t*, and the outer radius of the MT layer is *ρ*_2_ = *k*_0_*a*_2_ where *a*_2_ = *a*_1_ + *l*. The refractive index of the system is given by

$$n\left(\rho \right)=\{\begin{array}{cc} \begin{array}{c} n_{0}\\ n_{1} \end{array} & \begin{array}{c} 0\leq \rho <\rho_{0}\\ \rho_{0}\leq \rho <\rho_{1} \end{array}\\ \begin{array}{c} n_{2}\left(\rho \right)\\ n_{a} \end{array} & \begin{array}{c} \rho_{1}\leq \rho <\rho_{2}\\ \rho_{2}\leq \rho \end{array} \end{array}$$

with

$$n_{2}\left(\rho \right)=n_{a}+\Delta n\;\frac{a_{1}^{2}}{r^{2}}=n_{a}+\Delta n\;\frac{\rho_{1}^{2}}{\rho^{2}}$$

Let

$$\begin{array}{cc} \begin{array}{cc} \beta^{\left(1\right)}=1, & \tau_{0}^{\left(1\right)}={\left(n_{0}/n_{1}\right)}^{2}, \end{array} & \begin{array}{cc} \tau_{1}^{\left(1\right)}={\left(n_{1}/n_{2}\left(\rho_{1}\right)\right)}^{2}, & \tau_{2}^{\left(1\right)}={\left(n_{2}\left(\rho_{2}\right)/n_{a}\right)}^{2} \end{array} \end{array}$$

and

$$\begin{array}{cc} \beta^{\left(2\right)}=0, & \tau_{0}^{\left(2\right)}=\tau_{1}^{\left(2\right)}=\tau_{2}^{\left(2\right)}=1 \end{array}$$

where superscripts (1) and (2) denote transverse magnetic (TM) and transverse electric (TE) modes, respectively. This allows us to express the differential equation whose solutions can be used to construct the Debye potentials as

$$\frac{d^{2}Y_{m}^{\left(1,2\right)}}{d\rho^{2}}-\beta^{\left(1,2\right)}\frac{2}{n}\frac{dn}{d\rho }\frac{dY_{m}^{\left(1,2\right)}}{d\rho }+\left[n^{2}-\frac{m\left(m+1\right)}{\rho^{2}}\right]Y_{m}^{\left(1,2\right)}=0$$

where *m* = 1, 2, …. Except for *ρ*_1_ ≤ *ρ* < *ρ*_2_, solutions to the equation above are the Riccati-Bessel functions, which can be expressed in terms of the cylindrical Bessel functions of the first and second kind as

$$\psi_{m}\left(x\right)=\sqrt{\frac{\pi x}{2}}J_{m+1/2}\left(x\right)$$

$$\chi_{m}\left(x\right)=\sqrt{\frac{\pi x}{2}}Y_{m+1/2}\left(x\right)$$

$$\zeta_{m}\left(x\right)=\psi_{m}\left(x\right)+i\chi_{m}\left(x\right)$$

The two linearly independent solutions to the differential equation for the MT layer (*ρ*_1_ ≤ *ρ* < *ρ*_2_) are obtained using Mathematica’s NDSolve function. Using $u_{m}^{\left(1,2\right)}\left(\rho \right)$ and $v_{m}^{\left(1,2\right)}\left(\rho \right)$ to denote the two linearly independent solutions, we write $Y_{m}^{\left(1,2\right)}$ with yet-to-be-determined coefficients as

$$Y_{m}^{\left(1,2\right)}\left(\rho \right)=\frac{E_{m}}{n_{a}^{2}k_{0}^{2}}\{\begin{array}{cc} A_{0,m}^{\left(1,2\right)}\psi_{m}\left(n_{0}\rho \right) & 0\leq \rho <\rho_{0}\\ A_{1,m}^{\left(1,2\right)}\psi_{m}\left(n_{1}\rho \right)+B_{1,m}^{\left(1,2\right)}\zeta_{m}\left(n_{1}\rho \right) & \rho_{0}\leq \rho <\rho_{1}\\ A_{2,m}^{\left(1,2\right)}u_{m}^{\left(1,2\right)}\left(\rho \right)+B_{2,m}^{\left(1,2\right)}v_{m}^{\left(1,2\right)}\left(\rho \right) & \;\rho_{1}\leq \rho <\rho_{2}\\ -A_{m}^{\left(1,2\right)}n_{a}^{1-\beta }\zeta_{m}\left(n_{a}\rho \right)+n_{a}^{1-\beta }\psi_{m}\left(n_{a}\rho \right) & \;\rho_{2}\leq \rho \end{array}$$

where

$$E_{m}=i^{m+1}\frac{2m+1}{m\left(m+1\right)}E_{0}$$

and *E*_0_ is the electric field amplitude of the incident wave.

To solve for the coefficient $A_{m}^{\left(1,2\right)}$, which is associated with light scattered by the nanoparticle, we impose appropriate boundary conditions at *ρ* = *ρ*_0_, *ρ* = *ρ*_1_, and *ρ* = *ρ*_2_ and obtain

$$\begin{array}{l} \tau_{0}^{\left(1,2\right)}\left[A_{0,m}^{\left(1,2\right)}\psi_{m}\left(n_{0}\rho_{0}\right)\right]=A_{1,m}^{\left(1,2\right)}\psi_{m}\left(n_{1}\rho_{0}\right)+B_{1,m}^{\left(1,2\right)}\zeta_{m}\left(n_{1}\rho_{0}\right)\\ A_{0,m}^{\left(1,2\right)}\psi_{m}^{\prime }\left(n_{0}\rho_{0}\right)=A_{1,m}^{\left(1,2\right)}\psi_{m}^{\prime }\left(n_{1}\rho_{0}\right)+B_{1,m}^{\left(1,2\right)}\zeta_{m}^{\prime }\left(n_{1}\rho_{0}\right)\\ \tau_{1}^{\left(1,2\right)}\left[A_{1,m}^{\left(1,2\right)}\psi_{m}\left(n_{1}\rho_{1}\right)+B_{1,m}^{\left(1,2\right)}\zeta_{m}\left(n_{1}\rho_{1}\right)\right]=A_{2,m}^{\left(1,2\right)}u_{m}^{\left(1,2\right)}\left(\rho_{1}\right)+B_{2,m}^{\left(1,2\right)}v_{m}^{\left(1,2\right)}\left(\rho_{1}\right)\\ A_{1,m}^{\left(1,2\right)}\psi_{m}^{\prime }\left(n_{1}\rho_{1}\right)+B_{1,m}^{\left(1,2\right)}\zeta_{m}^{\prime }\left(n_{1}\rho_{1}\right)=A_{2,m}^{\left(1,2\right)}u_{m}^{{\left(1,2\right)}^{\prime }}\left(\rho_{1}\right)+B_{2,m}^{\left(1,2\right)}v_{m}^{{\left(1,2\right)}^{\prime }}\left(\rho_{1}\right)\\ \tau_{2}^{\left(1,2\right)}\left[A_{2,m}^{\left(1,2\right)}u_{m}^{\left(1,2\right)}\left(\rho_{2}\right)+B_{2,m}^{\left(1,2\right)}v_{m}^{\left(1,2\right)}\left(\rho_{2}\right)\right]=-A_{m}^{\left(1,2\right)}n_{a}^{1-\beta }\zeta_{m}\left(n_{a}\rho_{2}\right)+n_{a}^{1-\beta }\psi_{m}\left(n_{a}\rho_{2}\right)\\ A_{2,m}^{\left(1,2\right)}u_{m}^{{\left(1,2\right)}^{\prime }}\left(\rho_{2}\right)+B_{2,m}^{\left(1,2\right)}v_{m}^{{\left(1,2\right)}^{\prime }}\left(\rho_{2}\right)=-A_{m}^{\left(1,2\right)}n_{a}^{1-\beta }\zeta_{m}^{\prime }\left(n_{a}\rho_{2}\right)+n_{a}^{1-\beta }\psi_{m}^{\prime }\left(n_{a}\rho_{2}\right) \end{array}$$

The primes indicate differentiation with respect to *ρ*. These conditions ensure that the tangential components of the electric field and the magnetic field are continuous at the boundaries. We use Mathematica to solve the equations above for $A_{m}^{\left(1,2\right)}$ and calculate the extinction cross section numerically from

Qext=λ22πna2∑m=1M(2m+1)Re[Am(1)+Am(2)]

We truncate the series by choosing *M* to be at least as large as *x* + 4*x*^1/3^ + 2 where x = 2*πn_a_a*_2_/*λ* is the size parameter of the system [34,42].

To compute the extinction cross section of a simplified system without the intermediate layer, we set *t* = 0 (or equivalently, *ρ*_0_ = *ρ*_1_) and modify the expression for changed the expression for $Y_{m}^{\left(1,2\right)}$ to

$$Y_{m}^{\left(1,2\right)}\left(\rho \right)=\frac{E_{m}}{n_{a}^{2}k_{0}^{2}}\{\begin{array}{cc} A_{0,m}^{\left(1,2\right)}\psi_{m}\left(n_{0}\rho \right) & 0\leq \rho <\rho_{0}\\ A_{2,m}^{\left(1,2\right)}u_{m}^{\left(1,2\right)}\left(\rho \right)+B_{2,m}^{\left(1,2\right)}v_{m}^{\left(1,2\right)}\left(\rho \right) & \rho_{0}\leq \rho <\rho_{2}\\ -A_{m}^{\left(1,2\right)}n_{a}^{1-\beta }\zeta_{m}\left(n_{a}\rho \right)+n_{a}^{1-\beta }\psi_{m}\left(n_{a}\rho \right) & \rho_{2}\leq \rho \end{array}$$

We then follow the same procedures as above to determine $A_{m}^{\left(1,2\right)}$ and *Q*_ext_.
