# Supplementary material for: LSPR Biosensing Approach for the Detection of Microtubule Nucleation
Source: Sensors (Basel). 2019 Mar 23;19(6):1436. doi: 10.3390/s19061436 (PMC6471214; doi:10.3390/s19061436)
Supplement: Supplementary file 1 [file sensors-19-01436-s001.pdf]

Supplemental Materials

LSPR Biosensing Approach for the Detection of Microtubule Nucleation

Keisuke Hasegawa *, Otabek Nazarov and Evan Porter

Department of Physics, Grinnell College, 1116 Eighth Avenue, Grinnell, IA 50112, USA; nazarovo@grinnell.edu (O.N.); porterev17@grinnell.edu (E.P.)

* Correspondence: hasegawa@grinnell.edu; Tel.: +1-641-269-4024

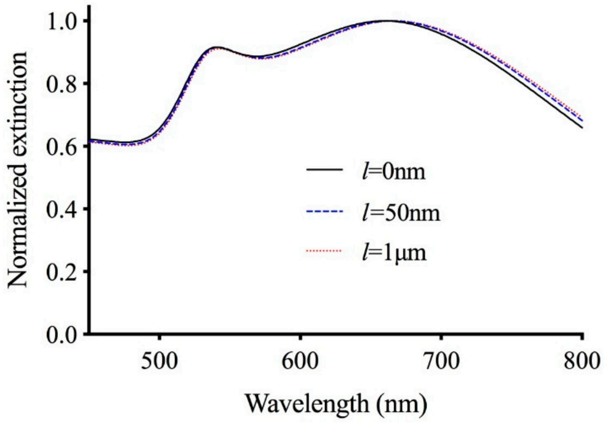

Figure S1. Calculated normalized extinction spectra for AuNPs with diameter $2a_0$ = 160nm, t = 0, and various MT layer thickness l. Peaks near λ = 660nm and λ = 540nm correspond to the dipole and quadrupole modes, respectively.

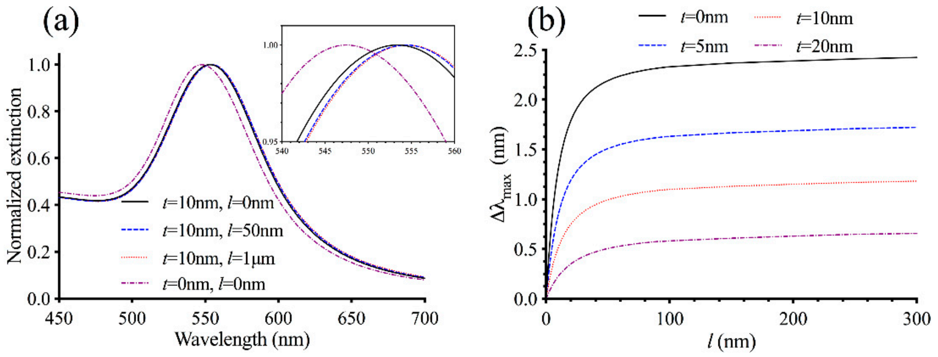

Figure S2. Calculated LSPR response of 80nm AuNPs with the intermediate layer and n_1 = 1.4. (a) Calculated normalized extinction spectra for AuNPs with diameter $2a_0$ = 80nm, intermediate layer with thickness t = 10nm and refractive index n_1 = 1.4, and various MT layer thickness l. The inset shows a magnified view of the spectra near their peaks. (b) The spectral shift $\Delta\lambda_{max} = \lambda_{max}(l) - \lambda_{max}(0)$ as a function of the MT layer thickness l for nanoparticles with different values for t.

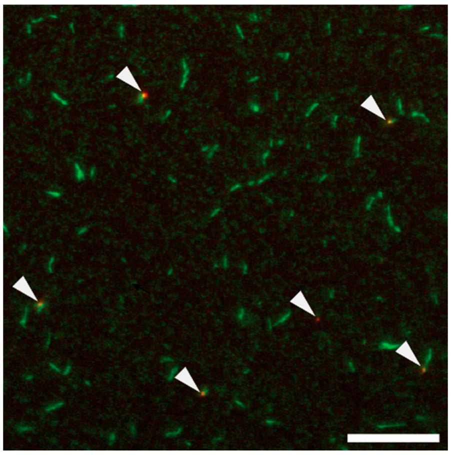

Figure S3. Fluorescence image of paclitaxel-stabilized sample after 1-minute incubation at 37°C. Represenative fluorescence image of biotin-PEG AuNPs decorated with neutravidin and ATTO655-streptavidin (red, also indicated with white arrowheads) and tubulin mixture containing biotinylated tubulin and Rhodamine-labeled tubulin (green) after a 1-minute incubation at 37°C in the presence of GMPCPP. Scale bar, 10μm..

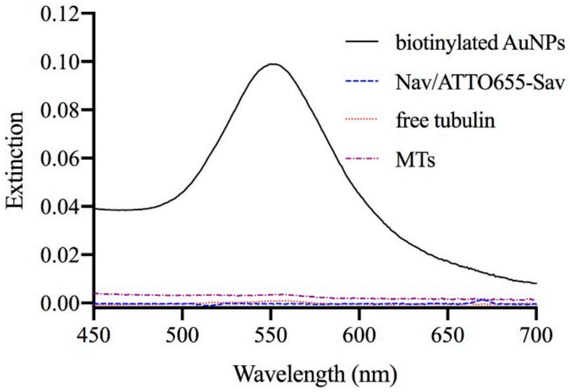

Figure S4. Comparison of the extinction spectra of biotin-PEG AuNPs, neutravidin (Nav) and ATTO655-streptavidin (Sav), free tubulin and MTs containing 4% Rhodamine-labeled tubulin.
